# Dysregulation of neuron differentiation in an autistic savant with exceptional memory

**DOI:** 10.1186/s13041-019-0507-7

**Published:** 2019-11-07

**Authors:** Jinjing Song, Xiujuan Yang, Ying Zhou, Lei Chen, Xu Zhang, Zhuxi Liu, Weibo Niu, Nengpeng Zhan, Xuelian Fan, Abdul Aziz Khan, Yifang Kuang, Lulu Song, Guang He, Weidong Li

**Affiliations:** 1 Bio-X Institutes, Key Laboratory for the Genetics of Development and Neuropsychiatric Disorders (Ministry of Education), Shanghai Key Laboratory of Psychotic Disorders, and Brain Science and Technology Research Center, Institute of Psychology and Behavioral Sciences, Shanghai Jiao Tong University, 800 Dongchuan Road, Shanghai, 200240 China; 2 Department of Psychiatry and Behavioral Sciences, Emory University School of Medicine, Atlanta, GA 30322 USA; 3 Department of Pediatric Surgery, Xin Hua Hospital, School of Medicine, Shanghai Jiao Tong University, Shanghai Institute for Pediatric Research, 1665 Kongjiang Road, Shanghai, 200092 China

**Keywords:** Autistic savant, Human induced pluripotent stem cells, TBR1, PAX6, FOXP2, Neurons

## Abstract

Autism spectrum disorder (ASD) is a heterogeneous group of complex neurodevelopmental disorders without a unique or definite underlying pathogenesis. Although savant syndrome is common in ASD, few models are available for studying the molecular and cellular mechanisms of this syndrome. In this study, we generated urinary induced pluripotent stem cells (UiPSCs) from a 13-year-old male autistic savant with exceptional memory. The UiPSC-derived neurons of the autistic savant exhibited upregulated expression levels of ASD genes/learning difficulty-related genes, namely PAX6, TBR1 and FOXP2, accompanied by hypertrophic neural somas, enlarged spines, reduced spine density, and an increased frequency of spontaneous excitatory postsynaptic currents. Although this study involved only a single patient and a single control because of the rarity of such cases, it provides the first autistic savant UiPSC model that elucidates the potential cellular mechanisms underlying the condition.

## Introduction

Autism spectrum disorder (ASD) is a constellation of early-onset neurodevelopmental disorders. It is characterized by social communication deficits and repetitive sensory-motor behaviors, and it is often accompanied by abnormalities in language development [1]. According to recent surveys, the prevalence of ASD among 8-year-old children in the United Sates was approximately 16.8 per 1000 children; the estimated prevalence in China was lower than those in other countries [2, 3]. ASD is a highly heritable mental disorder. Approximately 80% of cases of ASD have no clear etiopathology or related model, although many genetic variants have been identified in syndromic and non-syndromic ASD [4]. Many ASD risk gene variants have been linked to brain connectivity, the excitation-inhibition balance, and synaptic functioning [5–7]. According to reports on risk-genes, *TBR1* and five other genes, namely *CHD8*, *KYRK1A*, *GRIN2B*, *PTEN* and *TBL1XR1*, may contribute to 1% of sporadic ASD cases [8]. Recently, a group of 65 genes were identified with strongest statistical evidence, and this identification has expanded knowledge of the sets of highest-confidence risk genes associated with ASD [9]. Among these genes, *TBR1*—a putative transcription factor (TF)—is highly expressed in glutamatergic early-born cortical neurons; it dictates the expression of other risk genes, controls cortical development, and is implicated in intellectual disability [10–12]. *TBR1* expression requires *PAX6* [13–15], and *PAX6* is related to autistic behaviors and speech abnormalities [16, 17]. Following *TBR1* dictation, *FOXP2* is also related to some severe speech-language disorders and plays a role in cortical neurogenesis [18–21].

Savant syndrome is a condition in which prodigious talent can co-occur with developmental conditions [22]. In some cases of ASD, special abilities are accompanied by deficits; moreover, according to parental reports and psychometric tests, a third of adults with ASD exhibit savant skills in different domains [23]. Special isolated memory skills were the most frequently reported special abilities [24]. ASD children with special abilities exhibit more autistic traits, and multiple talent genes also influence the differences across individuals with ASD [25]. However, few models are available for studying the molecular and cellular pathogenesis of autistic savants. In the study of neurodevelopmental disease, the induced pluripotent stem cell (iPSC) approach has been particularly useful [26–29]. Human iPSC models can enable the analysis of neuronal phenotypes and the investigation of cellular mechanisms after the derivation of autistic savants’ somatic cells into neurons.

In this study, we generated a urinary iPSC (UiPSC) model of a 13-year-old autistic boy with a photographic memory and speech-language deficit. This idiopathic savant exhibited repetitive behaviors and impaired social communication. We discovered that compared with control neurons, upregulated transcription of ASD risk genes co-occurred with dysregulated cellular cortical development and synaptogenesis in the UiPSC-derived neurons of the autistic savant on day 42 after neural progenitor cells (NPCs) differentiation. Our study is the first to provide a UiPSC model of an autistic savant with a photographic memory.

## Results

### Generation of UiPSC-derived neurons of the autistic savant

**Fig. 1:**
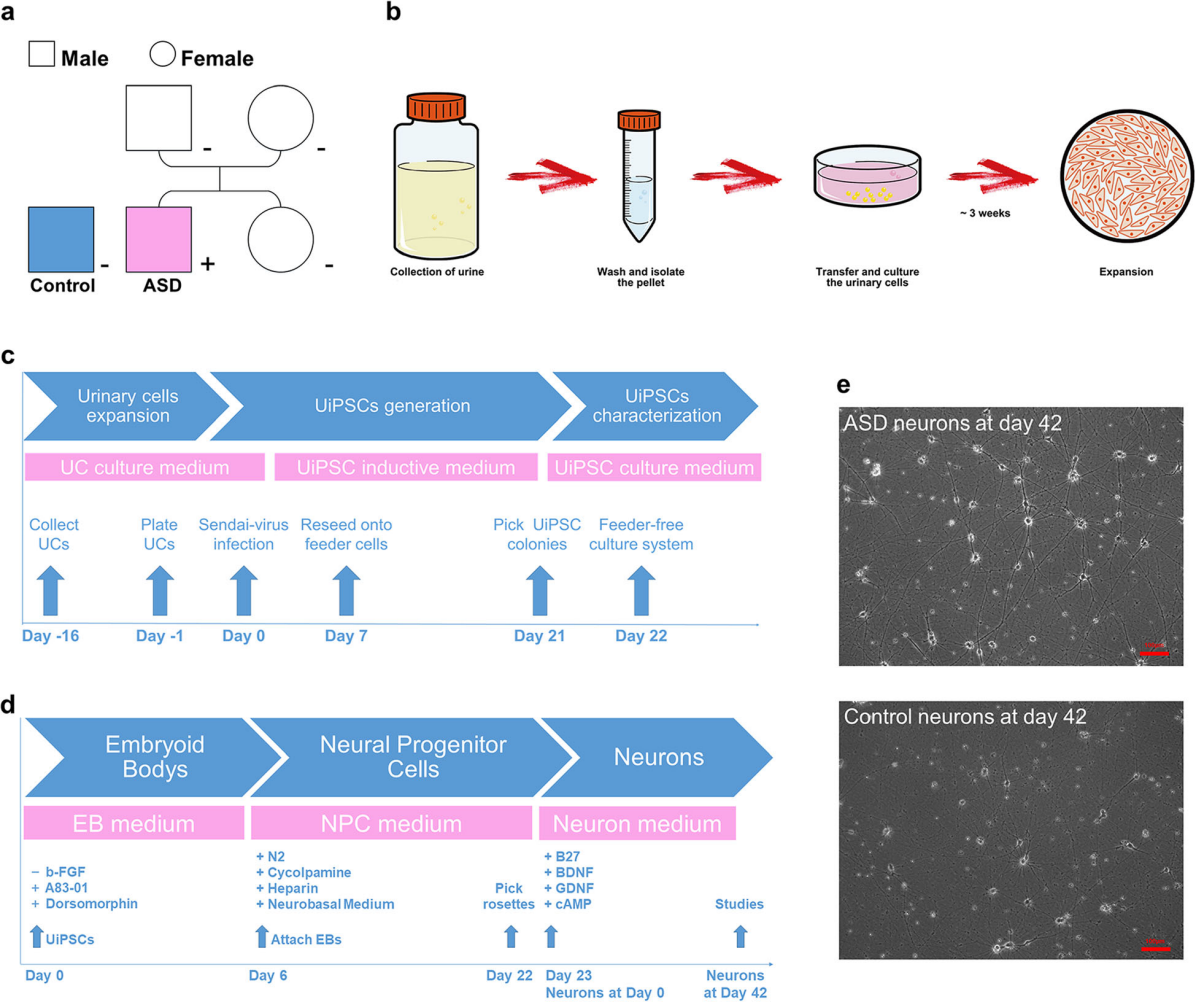
Modelling UiPSC-derived neurons. **a** All UiPSCs were generated from an autistic savant and an unrelated healthy individual as a control. The pink color indicates the autistic savant with photographic memory (ASD), whereas the blue color indicates the unrelated healthy control (Control). **b** Flowchart illustrating the collection, isolation, and expansion of urinary cells. **c** Flow diagram of UiPSCs generation. **d** Flowchart of neuron differentiation from UiPSCs. **e** Sample images of UiPSC-derived neurons without astrocyte co-culture on day 42 after NPCs differentiation. Scale bar, 100 μm. See also Fig. S1 and Table S1

Exfoliated renal epithelial cells were isolated from the urine of the autistic savant and an unrelated healthy control; the cells were then cultured for expansion (Fig. 1a and b and Additional file 3: Figure S1a). Approximately 3 weeks after isolation, urinary cells were replicated in sufficient quantity for the subsequent infection and exome sequencing studies (Additional file 1: Table S1). Attached urinary cells were infected using a Sendai-virus delivery system carrying the human OCT4, SOX2, KLF4, and c-MYC TFs; the urinary cells were then reprogramed into human UiPSCs (Fig. 1c and Additional file 3: Figure S1b). The advantages of this procedure have been described elsewhere [30–32]. We obtained three clone lines of UiPSCs from the autistic savant and two lines from the healthy control; all lines positively expressed pluripotent markers such as OCT4, NANOG, SOX2, SSEA4 and TRA-1-60 (Additional file 3: Figure S1c). All five UiPSC lines used in this study maintained a normal karyotype (Additional file 3: Figure S1d). The pluripotency of the UiPSC clones in vivo was confirmed by the generation of teratomas in severe combined immunodeficiency (SCID) mice. Furthermore, all UiPSC clones could generate teratomas that contained all three embryonic germ layers: endoderm, mesoderm and ectoderm (Additional file 3: Figure S1e). The results of teratomas generation proved that the five UiPSC clones could differentiate in vivo. Otherwise, positive alkaline phosphatase (AP) activities were also observed, confirming that all UiPSCs could maintain an undifferentiated proliferative state in vitro (Additional file 3: Figure S1f). These results indicated that the reprogramming of UiPSCs was successful.

The procedure for forebrain-specific neuronal differentiation is outlined in Fig. 1d. We initiated neuron differentiation by forming embryoid bodies (EBs). After approximately 3 weeks of differentiation, neural tube-like rosettes were manually collected, dissociated, and re-plated. All vital phases of NPC formation are illustrated in Additional file 3: Figure S1g. The UiPSC-derived NPCs were confirmed to positively express early neural precursor markers, such as nestin, SOX2 and musashi1 (Mui1) (Additional file 3: Figure S1g). After further neuron differentiation, the NPCs were differentiated into MAP2^+^ neurons with abundant expression of VGLUT1 (Additional file 4: Figure S2a), this observation is consistent with a previous description [27]. Six-week-differentiation constitutes a sufficient maturation process for detecting spontaneous synaptic activity in human iPSC-derived forebrain neurons [33]. In this study, we mainly focused on analyses of UiPSC-derived neurons on day 42 after NPCs differentiation (Fig. 1e).

### Upregulated expression of PAX6, TBR1, and FOXP2 in UiPSC-derived neurons of the autistic savant

**Fig. 2:**
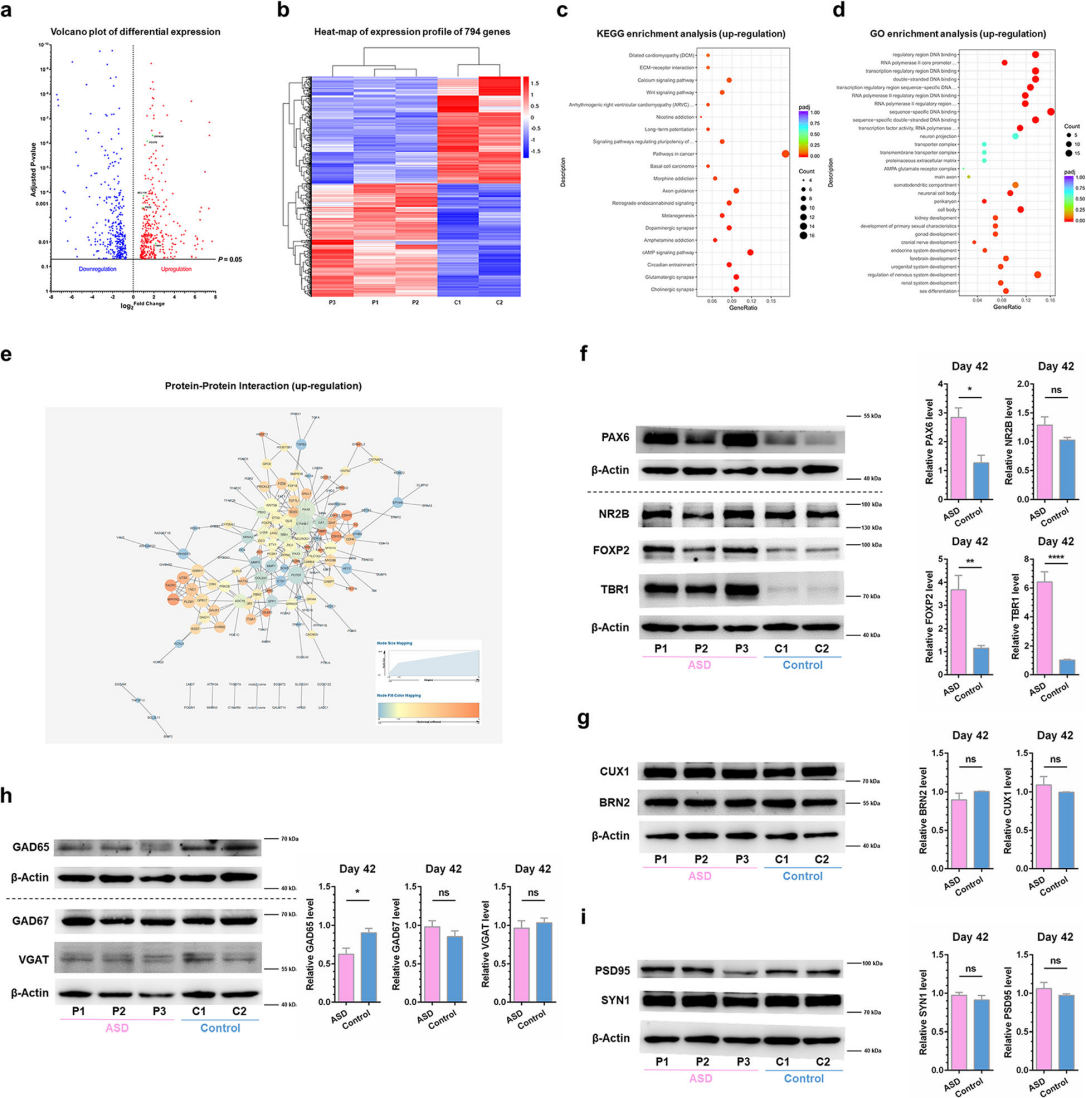
Dysregulated gene expression levels in UiPSC-derived neurons of the autistic savant on day 42. **a** and **b** DEG analyses of UiPSC-derived neurons without astrocyte co-culture on day 42. **a** Volcano plots of DGEs. The blue dots indicate downregulation, and the red dots indicate upregulation. In addition, the green dots represent major genes involved in the network of *TBR1*. **b** Heat-map analysis of DGEs. In total, 794 genes exhibited significant differential expression. **c**-**e** Enrichment analyses of upregulated DGEs of UiPSC-derived neurons. *Padj*, adjusted *p* value. Gene Ratio indicates the number of genes enriched in one pathway compared with the total genes changed in all pathways. Count indicates the number of genes. **c** KEGG enrichment analysis. **d** GO enrichment analysis. **e** PPI analysis. Degree was defined to range from 1 to 25 as the node size. A higher degree value indicates a more crucial gene, such as *CTNNB1*, *PAX6*, *WNT5B*, *TBR1*, *NEUROG1*, and *CDH10*. **f-i** Western blot analyses of ASD risk genes and specific neural markers. Sample Western blot analysis images and quantification are presented. Data were normalized to actin for sample loading and then normalized to C2 in the same blot for comparison. Values are presented as the mean ± SEM. *N* ≥ 3 cultures; unpaired Student’s *t* test. **f** PAX6, TBR1, and FOXP2 were upregulated in UiPSC-derived neurons of the autistic savant, whereas NR2B (*GRIN2B*) was not significantly different between the control neurons and the UiPSC-derived neurons of the autistic savant. Results of PAX6 were only from one culture. **g** CUX1 and BRN2, as the markers of layer II-V of the neocortex, were not significantly different between the two groups of neurons. **h** GAD65 was downregulated in UiPSC-derived neurons of the autistic savant, whereas GAD67 and VGAT were not significantly different between two groups of neurons. **i** PSD95 and synapsin1 (SYN1), as the synaptic proteins, were not different between the control neurons and the UiPSC-derived neurons of the autistic savant. See also Additional file 3: Figure S2 and Additional file 2: Table S2, Additional file 5: Table S3 and Additional file 6: Table S4

On day 42, we performed RNA sequencing (RNA-seq) analyses in UiPSC-derived neurons, without astrocyte co-culture, from all five clones. We detected 794 differentially expressed genes (DEGs), with 408 upregulations and 486 downregulations in the group of neurons from the autistic savant (Fig. 2a and b). Moreover, we observed an enrichment of upregulated pathways associated with mental disorders, such as the glutamatergic synapse pathway, Wnt signaling pathway, calcium signaling pathway and pathway of long-term potentiation, in the UiPSC-derived neurons of the autistic savant (Fig. 2c). An analysis of upregulated gene ontology (GO) enrichment revealed an abundance of genes associated with organ development, particularly neural system development, such as regulation of nervous system development, forebrain development, neurogenesis and negative regulation of nervous system development (Fig. 2d).

Among the aforementioned DEGs, we observed 55 upregulated TFs and 43 downregulated TFs in UiPSC-derived neurons of the autistic savant (Additional file 4: Figure S2b). High-confidence ASD risk genes, such as *PAX6*, *TBR1*, and *FOXP2* [7, 10, 19, 34, 35], were significantly upregulated in the UiPSC-derived neurons of the autistic savant (Fig. 2a and Additional file 4: Figure S2b). Both *PAX6* and *TBR1* constituted the vital TFs of the upregulated protein-protein interaction (PPI) network in the group of neurons from the autistic savant (Fig. 2e). Cortical connections are shaped by sequential maps of regional identity, propagated by the *PAX6*-*TBR1* TF cascade [11, 15]. Moreover, *TBR1* was reported to be a putative TF that was highly expressed in glutamatergic early-born cortical neurons [10] and was demonstrated to be a highest-confidence risk gene associated with ASD [8]. In the present study, Western blot analysis results revealed that TBR1 in the UiPSC-derived neurons of the autistic savant was upregulated by approximately six times compared with that in the neurons of the control (Fig. 2f). This finding was unexpected because most studies have reported many impaired conditions in *TBR1* in cases of ASD [7, 12, 18, 19, 34]. We also determined that PAX6 and FOXP2 in the UiPSC-derived neurons of the autistic savant were upregulated by approximately two and three times compared with those in the neurons from the control (Fig. 2f).

Although previous studies have revealed that NR2B (*GRIN2B*) expression was directly regulated by TBR1 [19, 36], we observed no significant differences in mRNA and protein expression levels between the groups (Additional file 2: Table S2 and Fig. 2f). Previous studies on iPSC-derived neurons have examined neuronal subtype differentiation by conducting quantitative analyses of specific neural markers for neurodevelopmental diseases [27, 29]. According to our RNA-seq analyses, markers II-V of the neocortex (*BCL11B*, *POU3F2*, *SATB2* and *CUX1*) and markers of glutamatergic neurons/GABAergic neurons (*SLC17A7* or *CAMK2A*/*GAD1 or SLC32A1)* did not differ between the two groups (Additional file 2: Table S2 and Additional file 6: Table S4). Our Western blot analysis results also showed that BRN2 (*POU3F2*), CUX1, GAD67 (GAD1), and VGAT (*SLC32A1)* in the UiPSC-derived neurons of the autistic savant were not different from those in controls (Fig. 2g and h). However, GAD65 was downregulated in the UiPSC-derived neurons of the autistic savant (Fig. 2h). A previous study demonstrated that TBR1 regulated the number of synapses in heterozygous mutant mice [7].. In the present study, we detected synapsin 1 (SYN1; a presynaptic marker) and PSD95 (a postsynaptic marker) expression in the UiPSC-derived neurons. Both SYN1 and PSD95 observed in the UiPSC-derived neurons of the autistic savant were not different from those observed in the neurons of the healthy control (Fig. 2i). This finding is consistent with the RNA-seq analysis results (Additional file 2: Table S2).

### Hypertrophic neural somas with enlarged spines and reduced spine density in UiPSC-derived neurons of the autistic savant

**Fig. 3:**
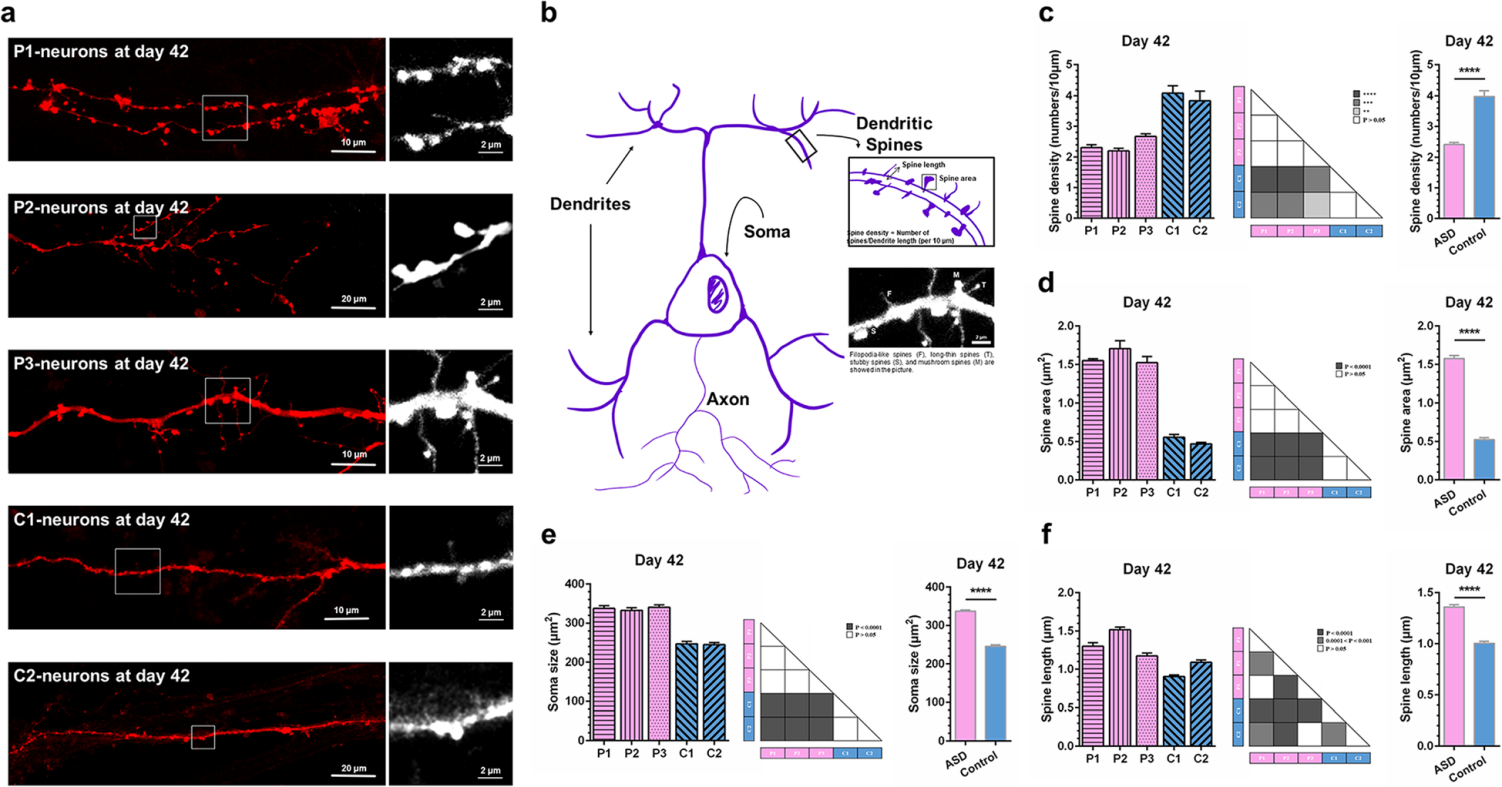
Hypertrophic neural somas with enlarged spines and reduced spine density in UiPSC-derived neurons of the autistic savant on day 42. **a** Sample confocal images of dendrites of UiPSC-derived neurons. Scale bar, 10 μm/20 μm/2 μm. **b** Hand-drawn diagram. The microstructure of a neuron, its classification, and the calculated formula of dendritic spines are presented. In addition, a sample image displays four types of spines, namely a filopodia-like spine (F), long-thin spine (T), stubby spine (S), and mushroom spine (M). Scale bar, 2 μm. **c-f** Quantification of neuronal morphological analyses. Values are presented as the mean ± SEM. *N* ≥ 3 cultures. ANOVA test and unpaired Student’s *t* test. “**” indicates 0.01 > *P* > 0.001. “***” indicates 0.001 > *P* > 0.0001. “****” indicates *P* < 0.0001. **c** Reduced average spine density in UiPSC-derived neurons of the autistic savant. **d** Enlarged average spine area in UiPSC-derived neurons of the autistic savant. **e** Increased average soma size in UiPSC-derived neurons of the autistic savant. **f** Increased average spine length in UiPSC-derived neurons of the autistic savant. Significant within-group differences were also observed. P2 was longer when compared with P1 and P3. In the control group neurons, C2 was longer when compared with C1

Previous studies have reported dysregulations of cellular phenotypes and the number of synapses in iPSC-derived neurons of idiopathic autistic individuals [28, 37, 38]. To investigate whether the UiPSC-derived neurons of the autistic savant had any morphological alterations when compared with the control neurons, we specifically labeled the membranes of the UiPSC-derived neurons with Dil by using a gene gun [39, 40]. The UiPSC-derived neurons, co-cultured on confluent astrocyte layers for 42 days after NPCs differentiation, were labeled with Dil and analyzed (Fig. 3a and b). Morphological analyses of the UiPSC-derived neurons of the autistic savant revealed that the dendritic spine density in these neurons was significantly reduced when compared with that in the control neurons (Fig. 3c). However, the average spine area and average spine length of the UiPSC-derived neurons of the autistic savant were greater than those of the control neurons (Fig. 3d and f). The average spine area of the UiPSC-derived neurons of the autistic savant was approximately three times larger than that of the control neurons (Fig. 3d). We measured the soma sizes of UiPSC-neurons without astrocyte co-culture on day 42. We conducted such measurements by analyzing bright-field images, and the analysis results showed that the average soma size of the UiPSC-derived neurons of the autistic savant was significantly larger than that of the control neurons (Fig. 3e). Overall, these data indicate the presence of hypertrophic neuronal somas with enlarged spines and reduced spine density in the UiPSC-derived neurons of the autistic savant.

### Increased frequency but not amplitude of spontaneous excitatory postsynaptic currents in UiPSC-derived neurons of the autistic savant

**Fig. 4:**
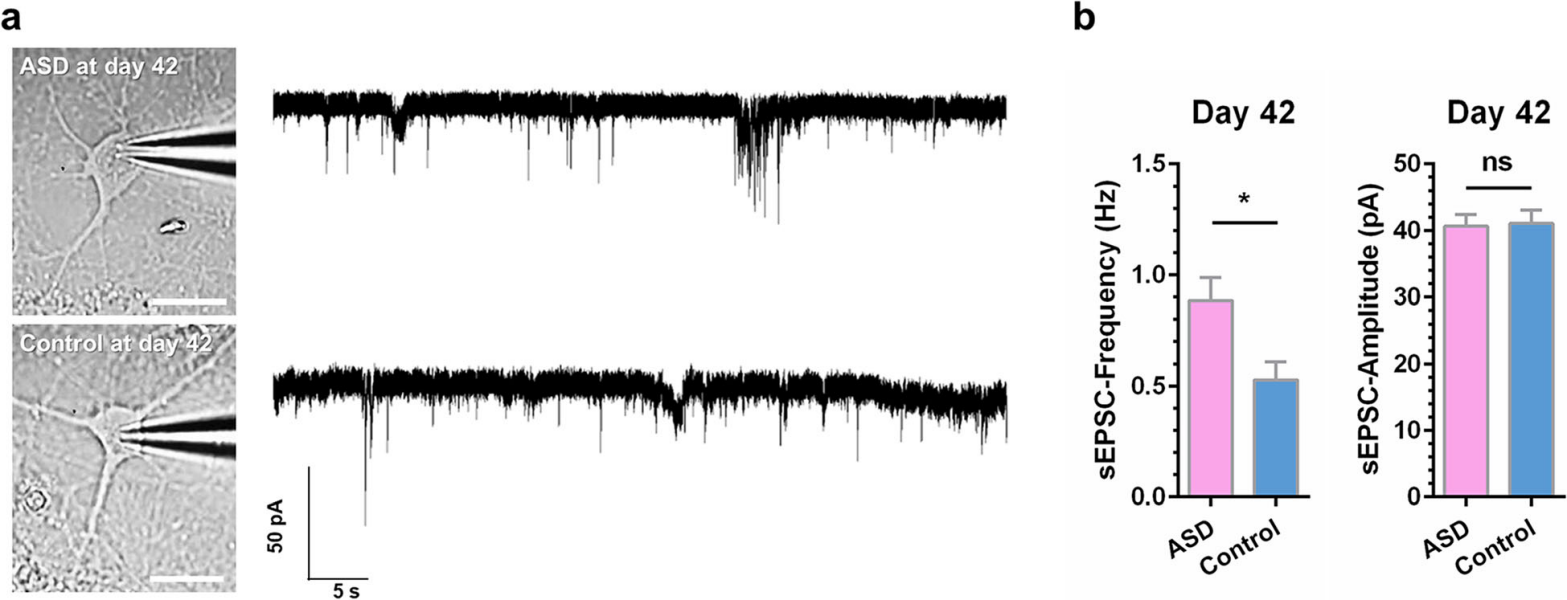
Increased frequency of sEPSCs in UiPSC-derived neurons of the autistic savant on day 42. **a** Sample images of UiPSC-derived neurons with astrocyte co-culture and sample whole-cell voltage-clamp recording traces of sEPSCs. Scale bar, 20 μm, 50 pA and 5 s. **b** Increased frequency, but not amplitude, of sEPSCs in UiPSC-derived neurons of the autistic savant. Quantification of sEPSCs. Values are presented as the mean ± SEM. *N* ≥ 4 cultures; *n* = 26–33 neurons for each line; unpaired Student’s *t* test

Previous studies on ASD have observed that the electrophysiological function of iPSC-derived neurons was dysregulated following cellular and synaptic morphological alternations [28, 37, 38]. In the present study, we conducted whole-cell recordings on UiPSC-derived neurons with astrocyte co-culture on day 42 after NPCs differentiation. The frequency, but not amplitude, of the spontaneous excitatory postsynaptic currents (sEPSCs) of the UiPSC-derived neurons of the autistic savant was significantly larger than that of the control neurons on day 42 (Fig. 4a and b). These electrophysiological results suggest a greater synaptic release probability in the excitatory synapses of the UiPSC-derived neurons of the autistic savant.

## Discussion

We studied an autistic savant with a photographic memory and speech-language deficit. Our study demonstrated that PAX6, TBR1, and FOXP2 (as vital TFs) were significantly upregulated in the UiPSC-derived neurons of the autistic savant when compared with the control neurons. In addition, our genomic data reveal that both the autistic savant and healthy control had amplifications in different loci of *PAX6*. No clear interpretations for the upregulation of PAX6 could be gleaned from the genomic data. This requires further investigation with regard to our models. Notably, in most previous reports on ASD and intellectual disability, *PAX6*, *TBR1* and *FOXP2* were either deficiently expressed or had lost functions [6, 8, 9, 16]. *TBR1* mutations have been implicated in impairments in social interaction, vocalization, memory and cognition [11, 12, 19]. *PAX6* and *FOXP2* have also been observed to be involved in the regulation of higher cognitive functions, including speech and language [16, 17, 41, 42]. In the present study, all of them were upregulated in the UiPSC-derived neurons of the autistic savant. According to studies on songbirds, FOXP2 is upregulated in a striatal region essential for song learning [43]. On the basis of our results, we could hypothesize that PAX6, TBR,1and FOXP2 upregulation explains the superior memory along with the speech-language deficit of the idiopathic autistic savant.

A decrease or increase in dendritic spine density is commonly observed in animal models of ASD-related genes, such as *Tbr1*, *Mecp2*, *Ube3a*, *Shank3*, *Tsc1*, *Fmr1*, and *Grin2b* mice [44]. In a mouse model, *Tbr1*, which is a potential master regulator in ASD, promotes synapse numbers through *Wnt7b* [7]. Knockdown of *FoxP2* reduces spine density in Area X of the zebra finch [45]. In mice carrying two alleles of the human *FOXP2* gene, dendrite length in the striatum increased [46]. The downstream *TBR1* target genes *CDH10*, *GPC6*, *LMO7*, and *CNTN2*, which encode membrane proteins that regulate cell adhesion and axonal outgrowth [47], were upregulated in the UiPSC-derived neurons of the autistic savant. The upregulation of these genes may explain the hypertrophic neural somas with enlarged spines and reduced spine density in the UiPSC-derived neurons of the autistic savant. To further explain the changes in soma size and dendritic spine density and size, studies on autistic savants regarding *TBR1* or *FOXP2* knockdown in UiPSC-derived neurons or overexpression of control neurons must be conducted in the future.

We did not observe a difference in the expression levels of *GAD1* and *SLC32A1*, which encode GAD67 and VGAT, between the two neuron groups in the RNA-seq analyses and Western blot analysis results, respectively. Notably, GAD65 levels were obviously decreased in the UiPSC-derived neurons of the autistic savant, although no difference in *GAD2* was observed in RNA-seq analyses. The analyses indicated the relationship between protein and mRNA levels under various scenarios, such as steady state, spatial and temporal variations of mRNAs, and local availability of resources for protein biosynthesis [48]. The mechanisms underlying decreased GAD65 are still unclear and require further investigations. Previous studies have determined that GAD65 and GAD67 differed with regard to their cellular and subcellular locations, transcription times, and functional purposes [49–51]. GAD65 is localized in nerve terminals and synthesizes GABA for neurotransmission in functions such as synaptogenesis and protection from neural injury [50, 52]. Moreover, GAD65 transcription occurs slightly later on in the development period when synaptic inhibition is more prevalent [50]. GAD65-synthesized GABA was reported to play a crucial role in the dynamic regulation of neural network excitability [53]. During neurotransmission, GAD65 forms a complex with VGAT; this helps to package GABA into vesicles for release [54]. Our results suggest a deficit of presynaptic inhibition in the synaptic release of the UiPSC-derived neurons of the autistic savant. This is a potential mechanism underlying the increased frequency of sEPSCs in the UiPSC-derived neurons of the autistic savant. The mechanism underlying the increased frequency of sEPSCs in the UiPSC-derived neurons of the autistic savant is not clear. The study showed that surviving TG (rTg(tauP301L)4510 tau mutant mice) cells had a significantly reduced total spine density but that the frequency of sEPSCs was increased, suggesting that during progressive tauopathy, cortical pyramidal cells compensate for the loss of afferent input with increased excitability [55]. The compensation might be the reason for the increased frequency of sEPSCs. However, further investigation is required on this topic, including recording mEPSCs, mIPSCs and sIPSCs parameters in the future.

Our results illustrated a possible link between dysregulated ASD genes, an aberrant cellular and synaptic morphology, and impaired electrophysiological function in cases of ASD accompanied by savant syndrome. Our UiPSC model of the autistic savant is useful for further research on the mechanisms underlying such conditions. Although this study generated UiPSC models of an autistic savant, it has some limitations that warrant consideration. The primary limitation is the inclusion of only one autistic savant and one control; it has been very hard to find other autistic children with a photographic memory and speech-language deficit. In addition, considering the high level of clonal variation in iPSCs, our study findings apply to only this autistic savant with a photographic memory and speech-language deficit.

## Methods

### The autistic savant subject

A 13-year-old male autistic savant with exceptional memory was selected from a Chinese SuperBrain Talent Pool. He was diagnosed as having ASD and a speech-language deficit by four hospitals when he was 6 years old. According to reports from his parents, his exceptional memory was noticed in that same year. We tested his memory when he was 13 years old. He exhibited exceptional recall upon quickly scanning the content of books that he had never seen previously.

### Collection and expansion of urinary cells

We established UiPSC models of a 12-year-old unrelated healthy control and a 13-year-old autistic savant with exceptional memory, both of whom were boys. The procedure and appearances are illustrated in Fig. 1b and Additional file 3: Figure S1a. We collected urine samples (approximately 100–400 mL) by using a sterile bottle containing a 5-mlL penicillin-streptomycin solution (Gibco, cat. no. 15070063). The samples were subsequently transferred to 50-mL centrifuge tubes and centrifuged at 1000 rpm for 10 min at room temperature. The pellets were collected into a single tube and washed with washing buffer, which was Dulbecco’s phosphate-buffered saline (DPBS, Gibco, cat. no. A1285601) supplemented with a penicillin-streptomycin solution. Urinary cells were resuspended in UC medium and transferred to a single well of a 12-well plate that had been coated with 0.1% gelatin (Millipore, cat. no. ES-006-B). The UC medium was a 1:1 mixture of complete Dulbecco’s modified Eagle’s medium (DMEM) containing DMEM/high glucose supplemented with 10% FBS and the renal epithelial cell growth medium (REGM; Lonza, cat. no. CC-3190). For each day during the first 4 days, we added 1 mL of UC medium. At approximately 96 h after plating, the urinary cells were removed all the medium, washed with DPBS, and added fresh UC medium. Half of the UC medium was replaced daily for approximately 10 days until the attached cells reached a density of 90%. Subsequently, the cells were passaged with TrypLE Select enzyme (Gibco, cat. no. 12563011) onto a 10-cm dish that was coated with gelatin for further expansion. This was considered passage 1 (P1). We continued to passage cells if required. The generation of UiPSCs from urinary cells at passages 1–3 was of a higher efficiency [30].

### UiPSCs generation

The protocol and appearances are illustrated in Fig. 1c and Additional file 3: Figure S1b. Urinary cells were plated in a gelatin-coated 6-well plate and incubated for 24 h. The cells were then infected using the CytoTune-iPS 2.0 Sendai Reprogramming Kit (Invitrogen, cat. no. A16517). The time point at which the cells were infected was considered day 0. After 24 h, Sendai virus-infected cells were replaced with fresh UC medium and changed every other day. On day 7, the infected cells were reseeded on the dishes that had been cultured primary mouse embryonic fibroblasts (Millipore, cat. no. PMEF-CFX) in UC medium. After 24 h of incubation, the infected cells were transferred from UC medium to UiPSC induction medium (UI medium). The UI medium comprised DMEM/F12, 20% KnockOut serum replacement (KSR; Gibco, cat. no. 10828028), 1% GlutaMAX (Gibco, cat. no. 35050061), 1% non-essential amino acid solution (NEAA; Gibco, cat. no. 11140050), 1% insulin-transferrin-selenium-ethanolamine (ITS-X; Gibco, cat. no. 51500056), 100 μM β-mercaptoethanol (β-Me; Sigma, cat. no. M3148), 50 ng/mL β-FGF (Peprotech, cat. no. 100-18B), 50 mg/mL ascorbic acid (Sigma, cat. no. A8960), 0.25 mM sodium butyrate (Sigma, cat. no. B5887), and 100 μg/mL Primocin (InvivoGen, cat. no. ant-pm-1). The reseeded cells were incubated for approximately 14 days, with the medium being replaced daily. After approximately 5 days of reseeding, the UiPSC colonies could emerge (Additional file 3: Figure S1b). When the colonies were ready for transfer, they could be picked and subsequently transferred to Matrigel-coated (Corning, cat. no. 354277) 24-well plates in UI medium. This was considered P1. The concentration of β-FGF was gradually decreased in the UiPSC culture medium at every passage until the UI medium contained 10 ng/mL β-FGF (UiPSC medium) at P5.

### Immunocytochemistry

UiPSCs were plated on the Matrigel-coated cell image dishes (Eppendorf, cat. no. 0030740.017) and incubated for 5 days. NPCs were plated on Matrigel-coated coverslips (Fisher Scientific, cat. no. 12–548-82) in the 12-well plates and incubated for approximately 7 days. The cells were fixed with 4% paraformaldehyde (PFA) for 15 min at room temperature, blocked with 0.25% Triton X-100 (Sigma, X100) and 10% goat serum in DPBS for 40 min at room temperature, and incubated with primary antibodies (Additional file 5: Table S3) at 4 °C overnight. They were subsequently incubated with secondary antibodies (Additional file 5: Table S3) for 1 h at room temperature and then mounted with antifade mountant with DAPI (Invitrogen, cat. no. P36935). Antibodies were prepared in 0.25% Triton X-100 and 10% goat serum in DPBS. Image were captured by a TCS SP8 STED 3X multiphoton confocal microscope (Leica).

### Karyotyping analyses

On day 5 after passaging, UiPSCs were treated in a colchicine solution (BBI Life Sciences, cat. no. A600322–0100) with a final concentration of 0.12 μg/mL for 1 h at 37 °C. Subsequently, the cells were dissociated with 0.05 mM EDTA and Trypsin mixed at a 9:1 ratio; the dissociated cells were then washed with DPBS. The pellet was gently resuspended with a 75 mM KCl solution and maintained in a 37 °C water bath for 20 min. We added 8–10 drops of a previously warmed fixative comprising methanol and acetic acid at a 3:1 ratio, after which we fixed the cells for 5 min at room temperature. After centrifugation, the pellet was replaced with a fresh fixative and fixed for 30 min at room temperature. The cells were then washed twice with a fixative and resuspended with 1 mL of fixative. Cell suspensions were sent for analysis to the Shanghai Da An Inspection for Medical Laboratory.

### Teratoma formation assays

UiPSCs were subcutaneously injected into the dorsal flank of SCID mice. Eight to 12 weeks after injection, the mice were monitored, and teratomas were dissected, fixed with 4% PFA in DPBS at 4 °C overnight, and gradually embedded in paraffin. Tissues were sectioned at 5 μm, followed by haematoxylin and eosin staining. Images were captured by a microscope (Nikon Eclipse 80i).

### AP activity in UiPSC assessment

On day 5 after passaging, UiPSCs were treated with an AP Detection Kit (Millipore, cat. no. SCR004) to determine AP activity. The UiPSCs were fixed with 4% PFA for 1–2 min at room temperature and washed with rinse buffer (20 Mm Tris-HCl solution with 0.05% Twwen-20, pH 7.4). Subsequently, the cells were replaced with stain solution and incubated in the dark at room temperature for 15 min. After incubation, they were washed again. Images were captured by a microscope (OPTIKA XDS-2).

### Neuron differentiation of UiPSCs

The procedure and appearances of UiPSC-derived neurons are illustrated in Fig. 1d and e and Additional file 3: Figure S1g. We differentiated the UiPSCs into forebrain-specific NPCs, as previously described [27]. The UiPSCs were detached with 1 mg/mL collagenase solution (Gibco, cat. no. 17104019) for 1 h at 37 °C; they were resuspended in EB medium—which was DMEM/F12 medium with 20% KSR, 1% GlutaMAX, 1% NEAA, 2 μM A 83–01 (Sigma, cat. no. SML0788), 2 μM Dorsomorphin (Sigma, cat. no. P5499), 100 μM β-Me and 100 μg/mL Primocin in not-treated polystyrene 6-well plates (Corning, cat. no. 3736)—for 6 days, with the medium being replaced daily. EBs were then replaced with NPC induction medium (NPC medium); this medium comprised DMEM/F12 and Neurobasal Medium (Gibco, cat. no. 21103049) mixed at 1:1 ratio, in addition to 1% NEAA, 1% N2 supplement (Gibco, cat. no. 17502048), 1% B27 supplement (Gibco, cat. no. 12587010), 2 μM cyclopamine (Sigma, cat. no. C4116), 2 μg/mL heparin (Sigma, cat.no. H3149), and 100 μg/mL Primocin. Subsequently, the EBs were transferred to Matrigel-coated 6-well plates to form neural tube-like rosettes. Rosettes were incubated for 16 days, with the NPC medium being replaced every other day. The rosettes were then picked and transferred to ultra-low-attachment-surfaced plates (Corning, cat. no. 3471) to form neural spheres in NPC medium. Alternatively, the rosettes were picked, dissociated with Accutase (Gibco, cat. no. A1110501) into single cells, and transferred to Matrigel-coated plates and culture slides in NPC medium. If they subsequently differentiated into neurons, NPCs were replaced with neuron medium (NM); the NM comprised Neurobasal Medium, 1% GlutaMAX, 1% NEAA, 1% N2, 1% B27, 10 ng/mL BDNF, 10 ng/mL GDNF, and 100 μg/mL Primocin after attachment. This was considered to be day 0 for the UiPSC-derived neurons. UiPSC-derived neurons were replaced weekly with half the culture medium. This occurred during continuous culturing and stopped when they were tested.

### RNA-seq analyses

On day 42, UiPSC-derived neurons without astrocyte co-culture were lysed with TRIZOL (Invitrogen) for gene expression analyses. The sample lysates of five lines were delivered at low temperature. RNA and library preparation, clustering, sequencing, and data analyses were performed by the Novogene Experimental Department. Sequencing libraries were generated using the NEBNext UltraTM RNA Library Prep Kit for Illumina (NEB) according to the manufacturer’s protocol; index codes were added to attribute sequences to each sample. After cluster generation, the library preparations were sequenced on an Illumina platform, and 125 bp/150 bp paired-end reads were generated. A differential expression analysis between the two groups was performed using the DESeq2 package in R (1.16.1). Genes with an adjusted *p* value of < 0.05 (obtained by DESeq2) were considered to be differentially expressed. A corrected *p* value of 0.05 and absolute fold change of 2 were set as the threshold for significantly differential expression. The KEGG is a database resource for understanding high-level functions and utilities of a biological system. Data were analyzed using the clusterProfiler package in R to test the statistical enrichment of DEGs in KEGG pathways. A GO enrichment analysis was implemented with the same package. A protein-protein interaction (PPI) analysis of DEGs was based on the STRING database. Otherwise, we used the Cytoscape (3.7.0) to construct the networks of the DEGs.

### Western blot analyses

On day 42, UiPSC-derived neurons were lysed with 250–300-μL ice-cold RIPA buffer (Sigma, cat. no. R0278) in each well of a 6-well plate; the RIPA buffer comprised a protease inhibitor and phosphatase inhibitor (Roche, cat. no. 4693132001 and cat. no. 4906837001). The cell lysis suspensions were incubated on ice for 30 min and then transferred to tubes. The UiPSC-derived neuron lysate was heated at 100 °C in a metal bath for 5 min; it was then immediately cooled in an ice bath. The sample matrixes were subsequently centrifuged at 12,000 *g* for 25 min at 4 °C. The protein lysate was aspirated, mixed with 5× loading buffer, dispensed into aliquots, and stored. The protein samples were electrophoresed and transferred to polyvinylidene fluoride (PVDF) membranes. The membranes were blocked with 5% BSA in Tris-buffered saline with Tween 20 (TBST; 0.05% Tween-20) for 2 h at room temperature, incubated overnight with primary antibodies (Additional file 5: Table S3) at 4 °C, incubated with secondary antibodies (Additional file 5: Table S3) for 1 h at room temperature, and washed with TBST (0.05% Tween-20). Antibodies were prepared in primary/secondary antibody dilution buffer (Beyotime Biotechnology, cat. no. P0256/P0258). Horseradish peroxidase signals were detected by a Tanon (5200) system. The derived images were analyzed using ImageJ (NIH).

### Preparation of primary astrocytes

Primary astrocytes were prepared from the cortices of Institute of Cancer Research (ICR) mice on postnatal day 0 (P_0_). P_0_ cortices were dissected, cut into small pieces, and dissociated with papain (Thermo Scientific, cat. no. 88285) in Hank’s balanced salt solution (HBSS) at 37 °C for 30 min. Cells were washed with HBSS and filtered, and epithelial cells were removed by differential time attachment. The cells were resuspended in astrocyte medium (AM) that comprised DMEM, 10% FBS, 1% NEAA, 1% GlutaMAX and Primocin, and they were incubated at 37 °C for 2 h. Subsequently, cell suspension was collected and transferred to T25 flasks that have been coated with 0.1 mg/mL poly-D-lysine (PDL; Sigma, cat. no. P0296). Confluent astrocytes were treated with 20 μM cytosine β-D-arabinofuranoside (AraC; Sigma, cat. no. C6646) for 72 h to further eliminate other cell types. A total of 2 × 10^5^ cells were seeded on PDL-coated tissue culture slides in 24-well plates with AM for further use in co-culture.

### Electrophysiological analyses

On days 41–43, patch clamp recordings from UiPSC-derived neurons with astrocyte co-culture were obtained at room temperature by using a Multiclamp 700B amplifier, Digidata 1550, and the corresponding acquisition software Clampex 10.3 from Molecular Devices (USA). Coverslips seeded with derived neurons were transferred into recording chambers and perfused continuously with an extracellular solution containing the following (in mM, all from Sigma-Aldrich): 128 NaCl, 5 KCl, 2 CaCl_2_, 1 MgCl_2_, 25 HEPES, and 10 glucose (added before use) with pH 7.4. All recordings were obtained with a glass patch electrode (3–6 MΩ tip resistance), which was filled with pipette solutions, in whole-cell recording mode and filtered at 1 kHz. In addition, sEPSCs were recorded for 5 min at a holding potential of − 60 mV by using Cs-based pipette solution containing the following (in mM, all from Sigma-Aldrich): 135 CsCl, 5 EGTA, 10 HEPES, 4 Mg-ATP, and 0.5 Na-GTP (pH 7.3). The series resistance was not compensated for but was monitored. Recordings with a > 25% change in series resistance were discarded. Data were analyzed using Clampfit 10.3 software.

### UiPSC-derived neurons labeled by gene gun

On day 42, UiPSC-derived neurons with astrocyte co-culture were washed with DPBS and rapidly fixed with 4% PFA for 1–2 min at room temperature. The UiPSC-derived neurons were removed from the fixative and specifically labeled with a Helios Gene Gun (Bio-Rad, 297BR). Bullets were prepared using Dil Stain (1,1′-Dioctadecyl-3,3,3′,3′-Tetramethylindocarbocyanine Perchlorate; Invitrogen, cat. no. D282). A slice with neurons was shot three times by one bullet. We added 100 μL DPBS to the neurons f to prevent them becoming dry, and we incubated the neurons for 3 h in the dark at room temperature. Slices were mounted with antifade mountant. The excitation and emission wavelengths of Dil (D282) were 549 and 565 nm, respectively. Images were captured by a TCS SP8 STED 3X multiphoton confocal microscope and were analyzed using Fiji software (ImageJ).

### Morphological structure of UiPSC-derived neurons analyses

We analyzed the morphological structure of the UiPSC-derived neurons; our analysis focused on spine density, spine area, spine length, and soma size. Illumination is presented in Fig. 3b. Images of neurons were captured and analyzed. Specifically, dendritic lengths were measured using a plug-in (Simple Neurite Tracer). Dendritic diameters, spine lengths, number of spines, and total areas of threshold (dendrite areas and spine areas) were measured using Fiji software. The average spine density was defined as the number of spines per 10 μm of dendritic length. The average spine area was defined as the total spine area divided by the number of spines. The area of total spines was defined as the difference between the total area of fluorescence and dendritic area. At least 950-μm-long dendrites from five or more neurons (from at least three cultures) were analyzed for each UiPSC line. Soma size was analyzed through ImageJ. At least 247 somas from three cultures without astrocyte co-culture were statistically analyzed for each UiPSC line.

### Quantification and statistical analysis

All experiments were replicated at least three times, and data from parallel cultures were acquired, except the wWestern blot of PAX6. GraphPad Prism (6.07) software was used for all statistical analyses. All individual data are presented as the mean ± standard error of the mean (SEM). Statistical significance was accepted at *p* < 0.05. We used Student’s unpaired *t* test to compare two groups. If five lines were compared, we used a one-way analysis of variance (ANOVA) with post hoc tests between groups corrected for multiple comparisons (Tukey). Statistical analyses and scale bars are described in each figure legend.

## Supplementary information

**Additional file 1:** **Table S1.** Exome sequencing of family members and control _ CNVs and SNPs. Related to Fig. 1.

**Additional file 2:** **Table S2.** Secondary RNA sequencing _ differentially expressed genes _ significant. Related to Fig. 2.

**Additional file 3:** **Figure S1.** Sample images of UiPSCs generation and NPCs differentiation. Related to Fig. 1.

**Additional file 4:** **Figure S2.** Other characters of UiPSC-derived neurons. Related to Fig. 2.

**Additional file 5:** **Table S3.** Antibody list. Related to Fig. 2.

**Additional file 6:** **Table S4.** Expression of marker genes of glutamatergic neurons and GABAergic neurons. Related to Fig. 2.

## Data Availability

The data that support the findings of this study are available from the corresponding author upon reasonable request.

## Abbreviations

AM: Astrocyte medium
ANOVA: One-way analysis of variance
AP: alkaline phosphatase
AraC: β-D-arabinofuranoside
ASD: Autism spectrum disorder
DEGs: Differentially expressed genes
DMEM: Dulbecco’s modified Eagle’s medium
DPBS: Dulbecco’s phosphate-buffered saline
EBs: Embryoid bodies
GO: Gene ontology
HBSS: Hank’s balanced salt solution
ICR: Institute of Cancer Research
iPSC: induced pluripotent stem cell
ITS-X: Insulin-transferrin-selenium-ethanolamine
KSR: Knock Out serum replacement
Mui1: Musashi1
NEAA: Non-essential amino acid solution
NM: Neuron medium
NPCs: Neural progenitor cells
P0: Postnatal day 0
P1: Passage 1;
PDL: Poly-D-lysine
PFA: Paraformaldehyde
PPI: Protein-protein interaction
PVDF: Polyvinylidene fluoride
REGM: Renal epithelial cell growth medium
RNA-seq: RNA-sequencing
SCID: Severe combined immunodeficiency
SEM: Standard error of the mean
sEPSCs: Spontaneous excitatory postsynaptic currents
SYN1: Synapsin 1
TBST: Tris-buffered saline with Tween 20
TF: Transcription factor
TG: rTg(tauP301l)4510 tau mutant mice
UI medium: UiPSC induction medium
UiPSCs: Urinary induced pluripotent stem cells
β-Me: β-mercaptoethanol
