# Supplementary material for: Dysregulation of neuron differentiation in an autistic savant with exceptional memory
Source: Mol Brain. 2019 Nov 7;12:91. doi: 10.1186/s13041-019-0507-7 (PMC6836402; doi:10.1186/s13041-019-0507-7)
Supplement: Supplementary file 3 — Additional file 3: Figure S1. Sample images of UiPSCs generation and NPCs differentiation. Related to Fig. 1. [file 13041_2019_507_MOESM3_ESM.pdf]

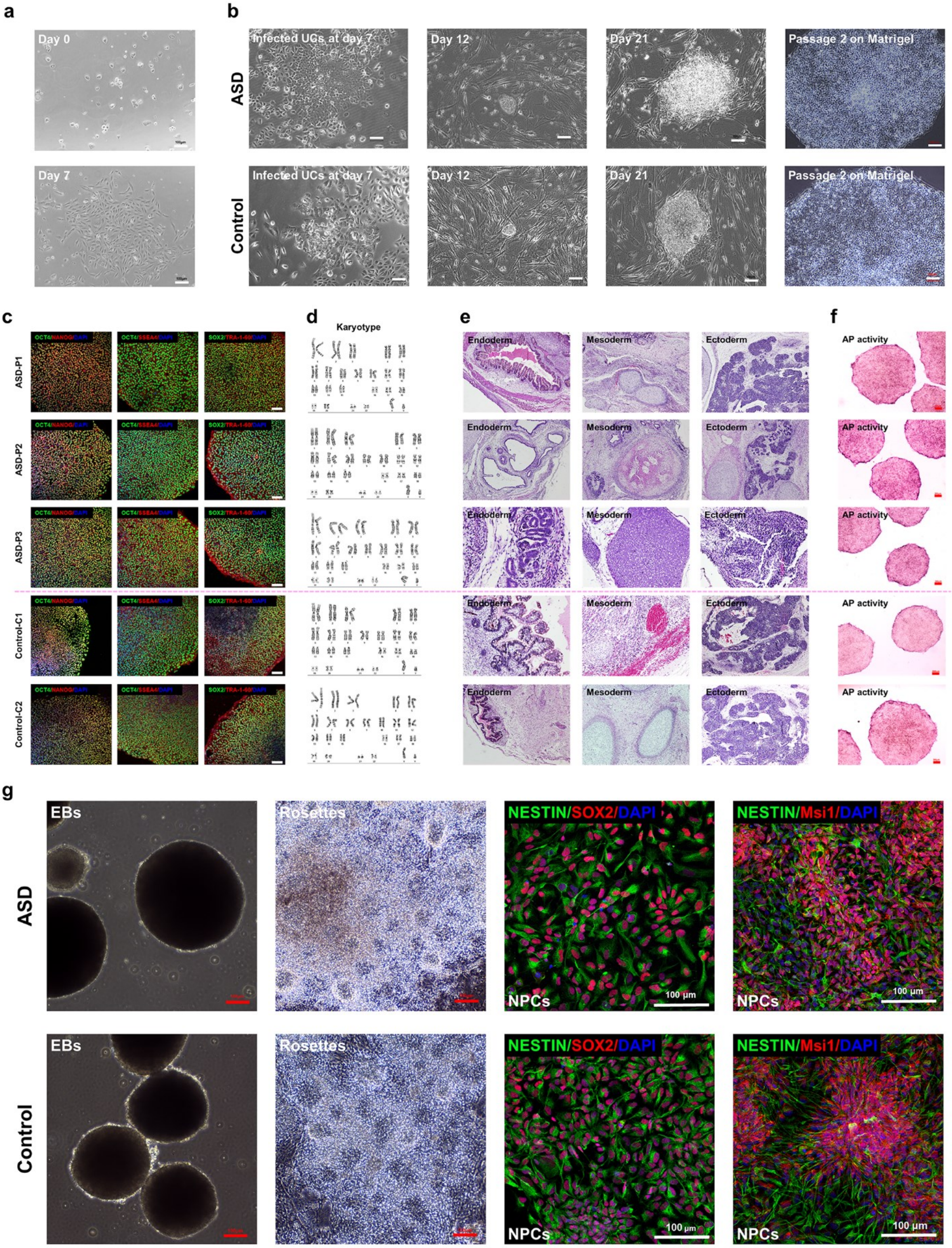
a
b
Day 0
Day 7
ASD
Control
Infected UCs at day 7
Day 12
Day 21
Passage 2 on Matrigel
c
d
Karyotype
e
f
ASD-P1
ASD-P2
ASD-P3
Control-C1
Control-C2
OCT4/NANOG/DAPI
OCT4/SSEA4/DAPI
SOX2/TRA-1-60/DAPI
Endoderm
Mesoderm
Ectoderm
AP activity
g
EBs
Rosettes
NESTIN/SOX2/DAPI
NESTIN/Msi1/DAPI
NPCs
100 μm

Additional file 3. Figure S1. Sample images of UiPSCs generation and NPCs differentiation. Related to Figure 1

(a) Sample images of urinary cells at the different phases after isolation. (b) Sample images of the different phases of Sendai virus-infected urinary cells, and sample images of picked UiPSCs cultured on Matrigel. (c-f) Characterization of five UiPSC lines. (c) Sample confocal images of immunostaining of pluripotency-associated markers for five UiPSC lines. Scale bar, 100 μm. (d) Sample images of normal karyotyping of all UiPSC lines. (e) Pluripotency of UiPSCs in vivo. Shown were sample images of cell types of three germ-layers in teratomas dissected from UiPSC-injected SCID mice. (f) Pluripotency of UiPSCs in vitro. Shown were sample images of positive staining for AP activity. Scale bar, 200 μm. (g) Sample images of three types of cells at different phases of neural differentiation. They were embryoid bodies (EBs), neural tube-like rosettes and dahlia-like neural progenitor cells (NPCs). The markers of NPCs were nestin, SOX2 and musashi1 (Msi1) at low and high cell densities. Scale bar, 100 μm.
