# Supplementary material for: Dysregulation of neuron differentiation in an autistic savant with exceptional memory
Source: Mol Brain. 2019 Nov 7;12:91. doi: 10.1186/s13041-019-0507-7 (PMC6836402; doi:10.1186/s13041-019-0507-7)
Supplement: Supplementary file 4 — Additional file 4: Figure S2. Other characters of UiPSC-derived neurons. Related to Fig. 2. [file 13041_2019_507_MOESM4_ESM.pdf]

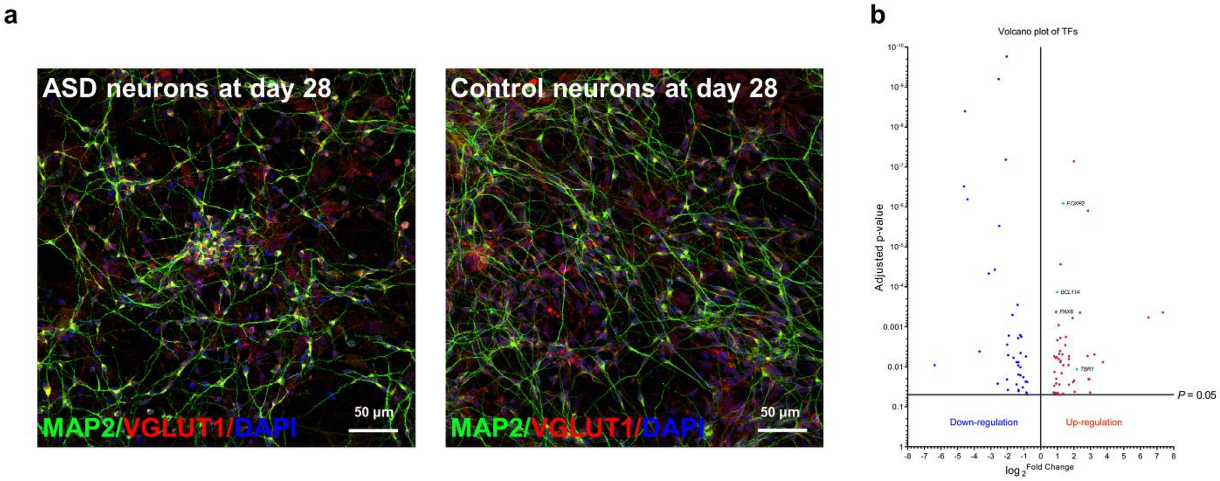

Additional file 4. Figure S2. Other characters of UiPSC-derived neurons. Related to Figure 2

(a) Sample images of UiPSC-derived neurons on day 28. Most UiPSC-derived neurons expressed MAP2 and VGLUT1 in two groups. Scale bar, 50 µm. (b) Volcano plots of differentially expressed TFs. The blue dots indicate downregulation, and the red dots indicate upregulation. In addition, the green dots represent major TFs involved in the network of *TBR1*.
