# Supplementary material for: Dysregulation of neuron differentiation in an autistic savant with exceptional memory
Source: Mol Brain. 2019 Nov 7;12:91. doi: 10.1186/s13041-019-0507-7 (PMC6836402; doi:10.1186/s13041-019-0507-7)
Supplement: Supplementary file 5 — Additional file 5: Table S3. Antibody list. Related to Fig. 2. [file 13041_2019_507_MOESM5_ESM.pdf]

Table S3 Antibody list

Primary Antibodies							
Marker Name	Host	ICC Dilution	MW(kDa)/(Dillution)	m/pAb	Species Reactivity	Vendor	Catalog#
Anti-SOX2	Rabbit	1/300	35 kD/(1/1000)	mAb	H	CST	3579S
Anti-TRA-1-60	Mouse	1/500	200-400 kD/(1/1000)	mAb	H	CST	4746S
Anti-OCT4	Rabbit	1/400	45 kD/(1/1000)	pAb	H	CST	2750S
Anti-SSEA4	Mouse	1/300	N/A	mAb	H	CST	4755S
Anti-NANOG	Mouse	1/1000	42 kD/(1/1000)	mAb	H	CST	4893S
Anti-NESTIN	Rabbit	1/400	220 kD/(1/2000-1/25000)	pAb	H	Millipore	ABD69
Anti-NESTIN	Mouse	1/100	220-240 kD/(1/1000-1/5000)	mAb	H	Millipore	MAB5326
Anti-Musashi 1	Rabbit	1/400	39 kD/(1/1000)	mAb	H, Ch, Quail	Abcam	ab178003
Anti-PAX6	Mouse	1/1000	46, 48 kD/(1/500)	mAb	H, M, R, Ch	Millipore	MAB5552
Anti-SOX1	Rabbit	1/200	40 kD/(1/200-1/1000)	pAb	H, M, R	Millipore	AB15766
Anti-MAP2	Rabbit	1/200	75, 82, 280 kD(1/1000)	pAb	H, M, R, Mk	CST	4542S
Anti-MAP2(2a+2b)	Mouse	1/800	280 kD/(1/800-1/2000)	mAb	H, M, R, B, …	SIGMA	M2320-100UL
Anti-VGLUT1	Mouse	1/1000	62 kD/(1/1000)	mAb	H, M, R	SIGMA	AMAB91041-100UL
Anti-GAD65	Mouse	N/A	65 kD/(1/1000)	mAb	H, M, R	Abcam	ab85866
Anti-GAD67	Mouse	1/400	67 kD/(1/500)	mAb	H, M, R	Millipore	MAB5406
Anti-GABA	Rabbit	1/400	N/A	pAb	A	SIGMA	A2052
Anti-GABA	Guinea Pig	1/500	N/A	pAb	A	Millipore	AB175
Anti-VGAT	Rabbit	1/100-1/1000	57 kD/(1/1000)	pAb	H, R, M, Mk	Synaptic Systems	131002
Anti-PSD95	Rabbit	1/200	95 kD/(1/1000)	mAb	H, M, R	CST	3450S
Anti-PSD95	Mouse	1/200	95 kD/(1/500)	mAb	H, M, R, B	Millipore	MAB1598
Anti-SYN1	Rabbit	1/200	77 kD/(1/1000)	mAb	H, M, R	CST	5297S
Anti-FOXP2	Rabbit	1/200-1/500	90 kD/(1/1000)	pAb	H	SIGMA	HPA000382-100UL
Anti-FOXP2	Rabbit	1/500-1/8000	~100 kD/(1/500)	pAb	H, M, R	Abcam	ab16046
Anti-TBR1	Rabbit	1/400	N/A	mAb	H, M, R	CST	49661
Anti-TBR1	Rabbit	1/200	74 kD/(1/500)	pAb	H, M, R	Abcam	ab31940
Anti-TBR2	Rabbit	1/100-1/500	72 kD/(1-200-1/1250)	pAb	H, M, R	Abcam	ab23345
Anti-BRN2(POU3F2)	Rabbit	1/3200	55 kD/(1/1000)	mAb	H, M, R	CST	12137S
Anti-CTIP2(BCL11B)	Mouse	1/50-1/500	116 kD/(1/200)	mAb	H, M, R	SANTA CRUZ	sc-365320
Anti-CUX1(CUTL1)	Mouse	1/50	~75 kD/(1/200)	mAb	H, M	Abcam	ab54583
Anti-p-NMDA Receptor 2A(GluN2A)	Rabbit	N/A	180 kD/(1/1000)	pAb	R, (H, M)	CST	4206S
Anti-NMDAR2A	Rabbit	N/A	165 kD/(1/1000)	mAb	H, M, R	Abcam	ab124913
Anti-p-NMDA Receptor 2B(GluN2B)	Rabbit	N/A	190 kD/(1/1000)	pAb	M, R, (H)	CST	5355S
Anti-NMDAR2B	Rabbit	1/100	166 kD/(1/500)	pAb	H, M, R, Ch	Abcam	ab65783
Anti-beta-Actin	Rabbit	N/A	45 kD/(1/1000)	pAb	H,M,R,Mk…	CST	4967S
Anti-GAPDH	Rabbit	N/A	37 kD/(1/1000)	mAb	H,M,R,Mk	CST	8884S
Secondary Antibodies							
Marker Name	Host	ICC Dilution	WB Dilution	m/pAb	Species Reactivity	Vendor	Catalog#
Anti-Mouse IgG, HRP	Goat	N/A	1/5000	pAb	Mouse	Invitrogen	A16006
Anti-Rabbit IgG, HRP	Goat	N/A	1/5000	pAb	Rabbit	Invitrogen	A16104
Anti-Mouse IgG, Alexa Fluor 594	Goat	1/1000	N/A	pAb	Mouse	Invitrogen	A11005
Anti-Rabbit IgG, Alexa Fluor 594	Goat	1/1000	N/A	pAb	Rabbit	Invitrogen	A11012
Anti-Mouse IgG, Alexa Fluor 488	Goat	1/1000	N/A	pAb	Mouse	Invitrogen	A11001
Anti-Rabbit IgG, Alexa Fluor 488	Goat	1/1000	N/A	pAb	Rabbit	Invitrogen	A11034
Anti-Guinea Pig IgG, Alexa Fluor 594	Goat	1/1000	N/A	pAb	Guinea Pig	Invitrogen	A11076
