# Supplementary material for: Dysregulation of neuron differentiation in an autistic savant with exceptional memory
Source: Mol Brain. 2019 Nov 7;12:91. doi: 10.1186/s13041-019-0507-7 (PMC6836402; doi:10.1186/s13041-019-0507-7)
Supplement: Supplementary file 6 — Additional file 6: Table S4. Expression of marker genes of glutamatergic neurons and GABAergic neurons. Related to Fig. 2. [file 13041_2019_507_MOESM6_ESM.pdf]

Table S4. Expression of marker genes of glutamatergic neurons and GABAergic neurons

Gene name	ASD	Control	$log_2^{Fold\ Change}$	*p* -value	Adjusted *p* -value
SLC17A7	23.781	16.297	0.543	0.469	0.938
CAMK2A	10.514	3.688	1.508	0.142	1.000
CAMK2B	59.205	67.040	-0.177	0.670	0.968
GAD1	153.019	201.184	-0.394	0.265	0.832
GAD2	6.015	22.164	-1.883	0.029	0.317
SLC32A1	1726.386	2596.795	-0.589	0.300	0.861
